# The Impact of Medical Tourism on Thai Private Hospital Management: Informing Hospital Policy

**DOI:** 10.5539/gjhs.v4n1p127

**Published:** 2012-01-01

**Authors:** Paul TJ James

**Affiliations:** Graduate School, Health Care Studies, Bangkok University Rama 4 Road, Klong-Toey, Bangkok, 10110, Thailand E-mail: paul.j@bu.ac.th

**Keywords:** Tourism, Medical, Private hospitals, Hospital policy, Bangkok

## Abstract

**Background:**

The purpose of this paper is to help consolidate and understand management perceptions and experiences of a targeted group (n=7) of Vice-Presidents of international Private Thai hospitals in Bangkok regarding medical tourism impacts.

**Methods:**

The method adopted uses a small-scale qualitative inquiry. Examines the on-going development and service management factors which contribute to the establishment and strengthening of relationships between international patients and hospital medical services provision. Develops a qualitative model that attempts to conceptualise the findings from a diverse range of management views into a framework of main (8) - Hospital Management; Hospital Processes; Hospital Technology; Quality Related; Communications; Personnel; Financial; and Patients; and consequent sub-themes (22).

**Results:**

Outcomes from small-scale qualitative inquiries cannot by design be taken outside of its topical arena. This inevitably indicates that more research of this kind needs to be carried out to understand this field more effectively. The evidence suggests that Private Thai hospital management have established views about what constitutes the impact of medical tourism on hospital policies and practices when hospital staff interact with international patients.

**Conclusions:**

As the private health service sector in Thailand continues to grow, future research is needed to help hospitals provide appropriate service patterns and appropriate medical products/services that meet international patient needs and aspirations. Highlights the increasing importance of the international consumer in Thailand’s health industry. This study provides insights of private health service providers in Bangkok by helping to understand more effectively health service quality environments, subsequent service provision, and the integrated development and impacts of new medical technology.

## 1. Introduction

South East Asia and especially Thailand is experiencing an expanding health services sector as hospital management – particularly private hospital management – have realised that offering world-class medical services can result in increased demand from overseas patients (Hazarika, 2010). This has helped private hospitals in Thailand increase their revenues by offering medical services at local premium costs, which are considerably cheaper than in many overseas countries. The Medical News (2009) reported that Thailand becomes Asia’s most popular medical hub with approximately 1.2 million medical tourists. However, this was the same total as in 2006 (Med-tourism-thailand, 2011) and in 2008 (Medical2, 2011). Although some forecasts suggest that this has been increased to 1.45 million medical tourists in 2010 (Medical Travel, 2011) with more than half of these going to one hospital (tourismthailand.org, 2011) – although, this hospital had only dealt with 420,000 patients in 2008 (Health-tourism, 2011). Another source recognises that there are perhaps 1.2 million international patients and speculate that 300,000 are medical tourists (Health-tourism, ibid). Consequently, the figures do not allow specific inferences to be made and that rather than a higher figure of 1.4 million, it is perhaps somewhat different and nearer 300,000 medical tourists that arrive in Thailand yearly. Nevertheless, based on these statements, it would seem difficult to know exactly where the medical tourism “dollar” is going in Thailand, as there are no government records published as to this particular purpose or to the revenue each hospital generates from this type of medo-socio-economic activity. As the Thai government does not keep data specifically about whether an individual comes to Thailand as a medical tourist except as a result of a pre-determined set of options (arrival card) – which does not visually include the term “medical tourist”. So the notion of Thailand as the most important medical hub (by numbers) cannot be substantiated, only assumed by NaRanong and NaRanong (2011) and maybe overhyped along with other countries such as Singapore or India. Without independent verification of actual medical tourism numbers then the industry is only speculating. Nonetheless, in some countries in SE Asia, a special medical visa has been developed (Smith, Chanda and Tangcharoensathien, 2009) which will help account for the numbers engaging directly in medical tourism. Given this, as there is a substantial difference in prices between the USA, UK and Canada and what is offered in Thailand, then there is the impression of a trend (Whittaker, Manderson, and Cartwright, 2010) that some tourists do come to Thailand with the express wish to undergo some kind of medical procedure which is the reason and exclusive focus of their visit. This raises the context for the first research question - What is it that patients want from their international health service provider?

For the purpose of this paper, medical tourism has been defined by Carrera and Bridges (2006a) as the organized travel outside one’s natural healthcare jurisdiction for the enhancement or restoration of the individual’s health through medical intervention. This has resulted in the “growing acceptance” of planned medical tourists (Chee, 2007) – those that have organised their procedures in advance. This is now perhaps a little limited as evidence suggests that there are those individuals who have found out while on holiday that such procedures are available at very affordable prices and quick turnaround times and so this definition must be widened to include ad-hoc patients. Subsequently, some tourists have found that they can combine holidays with medical procedures (Whittaker, 2008) at competitive prices, by providing extensive medical services with no-waiting and on-the-spot demands. In this respect, patients make choices because of their wealth, and are therefore not bound by the state, which often ordinarily provides the medical services for them at no cost. There are others whose circumstances reflect home-situations that are considered expensive, procedures that are often unavailable or have long-queues denoting their country’s poorer health service provision (Burkett, 2007). Whilst it can be said that many South East Asian countries offer such services, patients do not always want to take up medical procedures unless they are satisfied that the procedures are carried out in a safe environment and that there is low health risk following the procedure. Therefore, medical tourism suggests patients have choice in where to go for their medical needs and also that the private health insurance or publicly funded health provision in their country of origin does not cover these. In this respect, the patient is balancing the need to be prudent, conservative and safe with lower costs for medical procedures as well as having a choice of when those procedures are carried out and by whom. Often this means selecting where and with whom through a medical services broker or making selective individual decisions. Marketing promotion in home countries create demand for medical services overseas with individuals who have the money and time and where hospitals meet or exceed standards for patient safety and quality of care as they advertise their international accreditations. Whilst this paper indicates why medical tourism occurs, it is firmly targeted at individual medical professional responses at the service use stage, as very little research has been conducted in this area in SE Asia.

It is now common practice for private hospitals in Thailand to advertise and promote their medical services in developed countries as the cost in these countries of providing adequate and specific health increases. Consequently, the direction of medical travel is changing towards developing countries as patients seek faster and cheaper medical solutions (Carrera and Bridges, 2006a).

Private hospitals operating in Bangkok appear therefore to maximise their resultant profits by ignoring the lower to middle income populations that surround them, as no private hospital will treat anyone without the means to pay. Further, medical provision in Bangkok combined with the commercial operating notions of a 5 star hotel has led many first-world patients to select SE Asia as a medical-related destination. However, the impact of medical tourism has raised the question of whether medical tourism can improve the capability for local health services to enhance provision for the poor in SE Asia? (Blouin, 2010). The reverse rational can also suggest that as private hospitals are for-profit, then it is unlikely that in the short-term that health provision for the poor in Thailand will improve as local patients (middle-upper class) also seek medical assistance from such private hospital services Arunanondchai and Fink, 2007a). This raises the context for the second research question - In what ways do hospital services provide for international patient health requirements both in Thailand and overseas?

This paper draws on hospital management views of medical tourism and suggests the difficulty utilising the term for Thai private hospitals as it is perhaps a little too stereotypical just to accept the literature and denote that promotion through brochures, brokers and local/international websites can be used to gauge the numbers taking up such specialist medical activities across borders for cash. In this respect, very little research has been conducted to help condense management opinion regarding the issues involved in medical tourism and how these can be managed to provide a more effective understanding. Private hospitals by their very nature and focus appear to have major influence on hospital policy in general through their investment and operational orientation and thus appear to be a dominant force in Thai approaches to hospital development. This has helped the Thailand government to propose the development of Thailand - Bangkok - as a medical hub (MPH, 2003). This raises the context for the third research question - What are the reasons for the choices that management make to provide hospital services to medical tourists?

## 2. Methods

To gain a broader, deeper and more involved understanding of the issues generated within the Thai hospital management context and to consider more implicitly the issues and questions raised, this empirical groundwork utilised an interpretive approach (Walsh, White, and Young, 2008) to understand the perceptions of health service management regarding medical tourism. Hospital management were considered specialist knowledge agents as their opinions and experiences influenced policy application in the hospital. The research used a semi-structured questionnaire, which provided an appropriate element of context and flexibility (Cassell and Symon, 2004) and this further aided by applying an inductive/theory building approach (Glaser and Strauss, 1967). Given the lack of appropriately focused research in this area, this methodology is seen as suitable for creating contextual data for the purpose of forming richer theory development (Cayla and Eckhardt, 2007). The population for this study was managers of seven (7) international private hospitals located in Bangkok, Thailand - chosen through applying the approach of Carman (1990). After discussions with each hospital top management - VP-level managers would take part in the research and this reflected the criteria of theoretical purpose, relevance and appropriateness (Glaser and Strauss, 1967). It also indicated the importance given by the hospital to the research bearing. Using Glaser’s (2004) sampling processes, a total of seven VP-level managers were thus determined as the resultant sample frame, which was also considered convenience sampling by Harrel and Fors (1992).

Each interview was audio recorded for future analysis. Interviews were conducted in English and took approximately 50 minutes. All interviews were recorded digitally after gaining explicit permission, and were later transcribed verbatim using NVivo software. The conduct of the interviews follows a similar process used by Gray and Wilcox (1995), with each individual group being asked the same set of questions - modified through ancillary questioning (probes and follow-ups) in the same way as Balshem (1991). To increase the reliability of the data, the actual transcription was returned to each respondent – via e-mail - for correction, addition or deletion and return, which followed the process of validated referral (Reeves and Harper, 1981). Whole-process validity was achieved as the respondents were considered widely knowledgeable of the context and content associated with the research orientation (Tull and Hawkins, 1990).

Each interview was initially manually interrogated and coded initially using the EverNote software according to sub-themes that ’surfaced’ from the interview dialogue - using a form of open-coding derived from Glaser (1992a); and Straus and Corbin (1990). This treatment was also reinforced and extended through the use of thematic analysis conducted using the NVivo 9 - qualitative software package (Walsh *et al.*, 2008). Each interview was treated and coded independently. In this way, no portion of any interview dialogue was left uncoded and the overall outcome represented the shared respondents views and perspectives through an evolving coding-sequence (Buston, 1999). Various themes were sensed from the use of the software packages, as well as from the initial manual-coding attempts. This dual form of interrogation was an attempt to increase the validity of the choice of both key themes and sub-themes through a triangulation process. NVivo 9 was further used to explore these sub-themes by helping to pull together each of these sub-themes from all the interviews (Harwood and Garry, 2003). In this way, it was possible to capture each respondent’s comments across transcripts (Riessman, 1993) on each supported sub-theme and place them together for further consideration and analysis.

The structure of the outcome is greatly influenced by the emergence of the key-themes and sub-themes. The preferred strategy for the analysis of the primary data was to use the stated research questions, which are used as a guide to providing the outcome (based on Yin, 1994).

## 3. Results/Outcomes

### 3.1 Theme Outcomes

The various themes developed from the main interviews are presented in Table 1 below, and are essentially broken down into seven (8) key-themes: Hospital Management; Hospital Processes; Hospital Technology; Quality Related; Communications; Personnel; Financial; and Patients distributed across twenty-two (22) sub-themes. The placement of the sub-themes has been influenced by context of the key theme.

**Table 1:** Sub-theme observations

Major Theme	Sub-Themes						Total
Hospital Management	Reforms	11	Capacity	8	Emergency	4	23
Hospital Processes	Patient Data	14	Patient Support	12	Legal Issues	5	31
Hospital Technology	Medical Coverage	6	Costs	8	Tool	4	18
Quality Related	Accreditation	16	Auditing	11	QA	9	36
Communications	Co-ordination	7	Marketing Approaches	5			12
Personnel	Doctors	11	Nurses	9	Administration Staff	3	27
Financial	Costs	14	Revenue Cycle	7			21
Patients	Safety	8	Legal Issues	5	Medical Complication	3	16
Total		91		65		28	184

The outcomes are stated below where the discussion focuses on the sub-theme elements within each key theme and the subsequent impacts on Hospital Policy and are presented in Table 2, below. The discussion format used in this paper reflects the respondent’s voice through a streamlined and articulated approach for reporting. Consequently, the style adopted for reporting and illustrating the data is influenced by Gonzalez, (2008) and also Daniels *et al*. (2007) and is discussed below, focusing on the raised research questions and the resultant main themes.

**Table 2:** Summary of Research Questions and Major Themes that Inform Hospital Policy

Research Question	Major Themes	Informing Hospital Policy
Q1. What is it that patients want from their international health service provider?	Hospital Management	Experienced core doctors and nurses
		Appropriate Range of Medical Service Provision
	Quality Related	Certificated to appropriate standards
	Hospital Technology	Upto date technology
	Finance	Range of financial offerings - related to home credit-cards and banks
Q2. In what ways do hospital services provide for international health requirements both in Thailand and overseas?	Hospital Processes	Appropriate Range of Medical Service Provision
	Patients	Patients receive comparative 5 * hotel services
	Communications	Utilise all available media channels, using appropriate language and personal support mechanisms
	Hospital Technology	Technology diversity and scope
Q3. What are the reasons for the choices that management make to provide hospital services to medical tourists?	Finance	Assets management
		Range of financial offerings - related to home credit-cards and banks
	Personnel	Capability, training and availability of experienced staff

**Figure 1:**
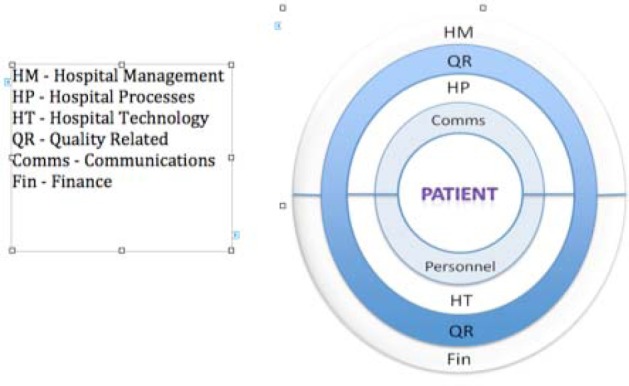
Model of Main Themes

Table 1, below, also shows the breadth of respondent illustrations/extractions as used in the reporting of this research. Figure 1, above shows a model of the managerial perspective with the main themes centred around the patient.

Q1 - What is it that patients want from their international health service provider?

Main Theme – Hospital Management

Hospital management appeared to recognise that reforms were necessary as changes were perceived to be required not only in the organisation but also in terms of staff development. As one respondent (M3) indicated we had to change. We needed to fundamentally change our whole outlook. Another respondent (M7) suggested we couldn’t be the same as everyone else. So, it was a case of focusing all our staff - doctors, nurses, everybody including the staff who carried patient’s belongings.

The introduction of newer and very expensive medical technologies had an impact. As one respondent (M1) suggested that it appeared to indicate that every member of staff was expected to learn new things, new ways of doing their job and on top of this was rebranding developments that appeared to expect changes in how staff (from every department) was expected to learn how to meet and greet patients from many countries. Another respondent (M5) supported this and stated with the new technology it was no longer seen as a Thai hospital but an international hospital in a Thai setting. For many hospitals, reforms included, for example as one respondent (M2) indicated internal training for doctors and nurses - who were given training in meeting patient needs, basic-language training - relevant to doctors orientation and more in-depth training for nurses who were expected to meet patients from overseas. Hospital reforms appeared to be more thorough for some as the balance between home-based patients and overseas patients changed, as depicted by another respondent (M6) who suggested that further changes at this hospital included training for receptionist nurses and other frontline medical staff - emergency staff and pharmacists who would explain medication as required. Most hospitals changed their policy on the the Case-Doctor, as one respondent stated each patient was assigned a doctor who would take charge of the medical provision throughout a patients stay together with an assigned international hospital representative who would also speak the patients language fluently.

An issue raised was that regarding core personnel shortages that were seen as inevitable - especially trained doctors in highly specialised disciplines. As one respondent (M3) illustrated this required some creativity, and here the hospital uses a radical form of knowledge sharing through the use of mobile technology - especially in an emergency situation. However, another respondent (M5) suggested that in more normal situations, doctors were shared among hospitals and were driven between appointments and calls by the network of hospitals This appeared to be a change to normal public hospital routines, but as stated by another respondent (M2) was used to ensure that overseas patients were given the attendance as required by their medical conditions.

In terms of hospital capacity, one respondent (M4) indicated that we have never been at full capacity, not even during emergencies. We have enough room to cater for local and international patients including our personnel. Another respondent (M3) suggested that we observe about 50% capacity to 60% - never much more than this. In terms of being private hospitals, the socio-economic profile of the patient would mean a mid to higher income group. Consequently, the expected behaviour of these individuals is that, as one respondent (M6) suggested they would not wait for medical services and expect a personal service delivery and further these two aspects create the focus for our health service delivery.

In terms of Emergency and Disaster preparedness/emergency protocols and teams private hospitals appeared very well prepared. For example, one respondent (M1) stated we have teams of professionals who can move at a moment’s notice. This includes the most sophisticated road ambulances and also our helicopter flying doctor service. We can be called to an emergency anywhere within 350 km of Bangkok. However, these are not normal and are used sparingly – and they are fairly expensive, as each trip has to be paid for by the patient or at least guaranteed by the patient through the banking system or through insurance. In many respects, these types of emergency services appear to be a luxury that not many patients can afford. One respondent (M3) indicated that these services are an essential addition to our medical services. We accept that it needs to be paid for, but we see this development as part of a managerial focus that will eventually have an impact on public health services too. Another respondent suggested that if there is a demand for these emergency services then we have to provide it. It saves lives and helps stabilise our communities.

Main Theme – Hospital Processes

Many of the hospitals “fail” to keep records of whether patients are medical tourists or are say expats. As one respondent (M7) indicated we are here to help patients with their medical problems. It doesn’t matter where they come from. Another respondent (M5) suggested that we do not really know whether a patient is a medical tourist or not. However, if a client contacts us before they arrive in the country, we will always do our best to accommodate them – especially if they are on a short-timeframe or if they want multi-procedures carried out at the same time.

Patient support and patient knowledge was raised as a necessary issue. As one respondent (M3) indicated we like our patients to make informed choices. We also want to help the patient professionally and therefore we need as much information as possible to advise them. Another respondent (M4) illustrated this as we help the patient make proper choices – at home, and in the hospital through the use of IT technology. We also can contact the patients’ doctor to quickly inform them of medical outcomes. Another respondent (M2) suggested that we try to get as much information from the patient before they leave their home. Otherwise they may have to bring that information with them. We only seek a discussion with the patient’s home doctor – if it’s necessary and if they give us permission to do so. Sometimes this causes us problems – medically and socially. A comment by another respondent (M1) asked does it really matter if they are tourists? However, from a government point of view this may illustrate an area for development because of the additional service GDP that is created, raising the notion that maybe the level of medical tourism is actually higher than accounted for at present.

Main Theme – Hospital Technology

In terms of medical coverage technology at all private hospitals in this study appears to be contemporary, modern and upto date. As one respondent (M7) indicated oh, yes. We try very hard to ensure we have the latest technology for diagnosing and hopefully confirming prognosis. However, the technology is used as a tool, rather than a singular determinator of evidence. As another respondent (M2) suggested technology is used almost everywhere, but our doctors have a lot of experience, we use the technology to confirm. We don’t use the technology to predict. Different points of view were raised regarding the use of technology such as one respondent (M3) who shared. I like the fact that we have technology available, but it isn’t always necessary, but it is available - especially if a patient wants it as a record for the future or to take home with them. Another respondent (M1) suggested that it’s important we have this backup. We can’t always be sure, so it’s important for our own development as well as the patient. Further, another respondent (M6) stated that sometimes patients seem a little more reassured when we use the technology, say MRI Scan, which often only confirms cheaper alternative of X-ray pictures.

The notion of cost related to technology was raised and this gave rise to a myriad number of responses. As one respondent (M4) indicated we have to bear the cost of very expensive technologies. Another respondent suggested that yes, very costly. Sometimes the technology just sits there not being used. So, it is very expensive. This aspect was confirmed by another respondent (M1) who stated unfortunately, we either invest or patients don’t come here again. We see technology as a way to differentiate this hospital from other hospitals. We either get the technology or our patients become dissatisfied and go elsewhere. On this point another respondent (M5) suggested that we see technology as a means to help our patients very quickly. This is one of the reasons why they come to us.

Private hospitals appear to be utilised at the capacity rate of 60% or less. However, ordinary everyday costs, as one respondent (M7) suggested gives us some problems. We have to manage our costs. There are always unexpected changes and these can affect our general and specific cost structures. However, this does not affect the patient, as another respondent (M2) indicated but when it directly affects our patients we try to communicate with them and show how the costs are built up. If a patient has a fixed cost package, then we will absorb the extra costs – its part of doing business. This is further explained by another respondent (M5) who suggested that when the patient undergoes more than one defined procedure then it can be difficult to precisely cost the outcome before we conduct the work. We always try to give patients an idea, but that isn’t enough sometimes – especially if they have come from overseas.

Respondents also showed that there were other costs issues there would Hidden costs – Unexpected charges; Unexpected Complications; Longer hospital stays; additional medicines and/or treatments/procedures; additional medicines after hospital treatment; inaccurate pricing estimates – before treatment; changes in medical conditions or new information about medical condition; preoperative tests/examination costs; and treatment delay

Main Theme – Quality Related

In terms of hospital accreditation, there would appear to an almost unanimous outcome. As one respondent (M1) stated it provides patients with trust in the hospital management - JCI accreditation, experienced American management, and the use of advanced technology. Another respondent (M6) suggested that our clients require that we are accredited. They can be secure in their mind that our professional judgement will have the client at the centre of what we do. This is further supported by another respondent (M4) who emphasised that accreditation is necessary for us to show both our domestic and international patients that we have the required quality in our systems and processes. It signifies an investment in the structure, processes and people in the hospital.

In terms of quality auditing the response was familiar, as one respondent (M3) stated each patient is given the opportunity to make comments and feedback about the medical services we give in terms of how we treat them etc. These are specifically to add to our processes and procedures that have been adopted for JCI and ISO 9000 accreditations. Another respondent (M1) indicated that we always carry out a service assessment before the patient leaves. It helps us manage for the future services we provide. In order to show how serious hospital management was, one respondent (M4) advised we are assessed on how well we adhere to the procedures and protocols we develop in order to get accredited. We train to make sure that nothing is left to chance. We have to get it right first time, every time.

Main Theme – Communications

Language was raised as a marketing tool. For example, one respondent (M5) indicated that 90% of our doctors speak English and about 45% of our nurses. We have a programme designed to help staff from all departments learn English, which is managed by a teaching group of native English speakers. It is an important part of the communication plan.

Co-ordination appeared to be an important facet of the patient communication strategy. This was seen as a simple measure but one that for overseas patients was a prominent requirement. As one respondent (M1) suggested we have a very good and well-tested system of communication that ensures that we can communicate with patients and their medical advisors while they are at home and supported by another respondent (M3) who stated each patient is allocated a member of the service staff who co-ordinates with the patient directly whether in Thailand or overseas, picks them up at the airport and ensures that everything is to their satisfaction. If they can’t solve any problem that’s raised then management can help solve any issue.

When the issue of marketing approaches was raised – Domestic and International - one respondent (M7) stated that we have to use many approaches today since there is a higher competition between hospitals – we know this. We have the philosophy that whatever medical procedures a patient requires, we make sure that they know exactly what it is that we will do and when and of course what the cost will be. Another respondent (M2) stated that we will talk directly with the patient in their home via phone, e-mail on videophone etc. This can mean that if surgery is required then a surgeon will talk directly to the patient – even if the patient is thousands of miles away.

Main Theme – Personnel

In terms of personnel, one respondent (M7) indicated that we try to employ the best doctors that we can. It’s not easy, as doctors can move from one hospital to another to get better working conditions. Another respondent (M1) stated that we do have a core of very qualified full-time staff, which meets with most needs internally. Sometimes we have to engage a doctor from a specialist field and they cost more – but our patients expect the best – and we always give our best at all times.

In terms of doctor’s experience and qualifications, one respondent (M2) stated that most of our doctors are trained in Thailand also internationally as this appears to help the reduction of the doctors leaving for overseas, as they remain better-paid professionals (Ramirez de Arellano, 2007). However, unlike public hospitals, one respondent (M4) stated quite clearly since all the doctors are trained, we do not have groups of young interns following a doctor while on his/her rounds. As such private hospitals are not considered training hospitals as this is reserved for public hospitals. This way we can give more personal attention, and also the patient sees the doctor for longer – and he’s always on call, if necessary.

Another respondent (M6) stated many doctors give their personal phone numbers to patients. This further creates a sense of bonding and trust between the doctor and the patient.

Main Theme – Financial

There would appear to be some financial challenges that private hospitals have to meet. For example, managing bad debts. As one respondent (M4) indicated as a doctor I will treat anyone who needs our help. However, as an administrator, we have to be very careful with costs and payments. Another respondent (M2) reiterated that although our priority is always to provide the highest standards in medical care, we have to be cautious with financial issues as some patients lack the funds to support their medical treatments. Given this, we have to behave just like a 5 star hotel, when registering the patient first, we will always take a copy of a credit-card/passport as additional insurance - even if they have validated insurance.

In terms of the revenue cycle, one respondent (M6) indicated that some patients have stays that last several months, but mostly it is less than 5 days. Consequently, there is often a delay of payment to the hospital - especially insurance companies. We recognise that this is the norm. Another respondent (M3) stated that it does create issues when on-going medical costs build-up and we have no cash remittance or revenue. In many cases we ask for partial payment – especially when the costs are likely to be in the hundreds of thousands and more than one procedure carried out. Thus it would seem that the revenue cycle isn’t as smooth as say in a manufacturing environment, but it is recognised by management that cycles have to be managed.

Main Theme – Patients

Under the theme patient safety, one respondent (M5) indicated that we are very careful in ensuring that patient safety is a top priority for all staff. For example, before a room is made available for a patient, it is serviced to a meticulous degree to reduce the risk of spreading any hospital-acquired infections. This is one of our major concerns. Another respondent (M1) suggested that at this hospital we have protocols in place that ensure staff, patients and visitors are only allowed in certain areas once they have been screened. On this important point, another respondent (M6) stated that we make sure that patient and staff safety are focused on even before we are fully aware of a patients medical needs.

When patient legal issues were raised, it became apparent that Thai hospitals had a strong standing as complaints were being made, but they were more of process related, rather than medical. For example, one respondent (M4) indicated that sometimes we have complaints, but these are very quickly sorted. These are often to do with being in strange surroundings. We learn how to deal with each patient, as each patient reacts differently. When an patient issue is raised that cannot be solved at the patient level, a wider response ensues. For example, one respondent (M2) indicated that we have protocols that are used to ensure that each patient is looked after very well. We also have protocols when the patient has an issue and we try to ensure that everyone involved in the patients well-being discusses the issues raised. This is what we call targeted patient action. Another respondent (M6) suggested that we don’t get many complaints, but sometimes the problems are not with the hospital at all. For example, it may involve special travel arrangements that we weren’t told about or an aircraft cancellation. However, we aim to ensure that complaints are reduced to a minimum and we do this by anticipating issues before they become complaints.

Hospital management appeared to be aware that patients were all different, and that unknown medical issues can arise during initial assessment or even during surgery. For example, one respondent (M1) indicated that sometimes, a client is weak or that the client wants a particular procedure and when sometimes we are doing this we find something else also. Another respondent (M7) stated it is difficult to manage because surgery is not like McDonald’s – we can’t always deliver what is ordered. This is because, each patient is different and we try to ensure that they get what they come here for and also that means getting value for money.

Finding new medical complications may also create new complaints and greater financial issues for the patient as there may be additional costs and changed timeframes associated with a given procedure. One respondent (M3) raised this issue, and stated that it is a difficult task to determine whether to carry out additional unintended surgery or leave it until the patient can make an informed choice. On this point another respondent (M4) suggested we can only do our best as professional doctors. We always make such decisions as a unified team in the best interests of the patient.

An issue was raised by many respondents in terms of emergency medical cover. This appeared to give doctors and management a challenge. For example, one respondent (M4) indicated that we are proud to provide emergency medical cover to anyone. But this is a business, and most patients pay straightaway. Sometimes though, it can be that we have to absorb some costs. That’s the medical industry – it happens everywhere. However, in most circumstances such emergencies are managed through the normal patient process. For example, one respondent (M2) suggested that for any patient, if there is an emergency or a patient wants to talk to his or her own doctor we often arrange that. Whether it’s the doctor assigned here or an overseas doctor, we try to accommodate that. We help our patients become as relaxed as possible so that they can recover quicker.

## 4. Discussion

**Figure 2:**
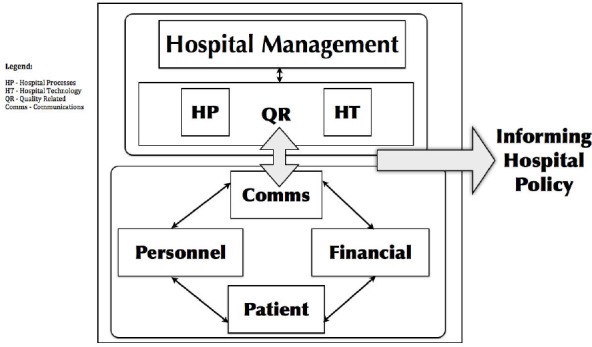
Model of Outcomes

Figure 2, below illustrates the derived relationships between the major themes informing hospital policy. The model further shows the conceptual development and relationships perceived to correspond to the features informing hospital policy which allows hospital management to focus on how these influence their strategic perceptions and intentions.

Hospital management appeared to make changes associated with the drive to offer foreign patients more facilities, often in better surroundings, guided by staff - nurses and doctors - and using contemporary medical technology that enhanced the working relationship between patients and their health service provider.

Private hospital policy appears to be driven by a contemporary management approach and strategy and this integrates with the patient through links in communications, personnel development and costs/financial arrangements. However, total integration of technology is seen as a barrier because of cost (technologyreview.com, 2009). This is explored further by taking each research question and teasing out the impacts of the related major themes on the main characteristics underpinning hospital policy. These characteristics include: Hospital Management, Quality Related, Hospital Technology, Finance, Hospital Processes, Patients, Communications, and Personnel.

Q1. What is it that patients want from their international health service provider?

The marketing practices of hospital management appeared to engage with patient requirements from first contact, to the conclusion and follow-up of patient outcomes. In this respect, much of the hospital running processes appeared to be developed and focused on providing a diversity of medical provision enhanced by the application of certified quality related processes that underpin the strategies for matching hospital technology and processes with the patient-doctor needs (Weinbroum, Ekstein and Tiberiu, 2003). Good leadership skills (Kizer, 2001) and business skills (Jennings et. al, 2007) therefore appeared to be an essential attribute of this type of hospital strategic engagement. Experienced core doctors and nurses with enhanced working relationships (Hughes, 2008) appear to be a characteristic that observable research outcomes could support resulting in a partnership in care situation (Overton-Brown and Anthony, 1998). In this respect, it would also appear that greater hospital experience would reveal more open communication tendencies and a more informative outlook that underpins the educational interactive-counselling approach (Prideaux *et al.*, 2001) used by doctors and nurses (Maguire, 1988). Hospital management also appeared to support the need for extending the range of medical service provision (Friedman *et al*. 2002), which in many circumstances created a larger hospital facility with medical technology utilisation at low levels consistent with developing support information systems required for managing elements of risk and moderating care (Kohn 2000). This appeared to be a fundamental management decision to show that the hospital could accommodate whatever medical plans patients would need and to act as a one-stop medical facility (Kohn 2000) which also impacted on hospital competitiveness (Robinson, 2001) with possible unavoidable duplication (Trinh, Begun and Luke, 2008) of medical services. This may also lead to the creation of additional cost (Lee and Stein, 1980) and reduce overall hospital industry efficiency. Some hospital management do not see this as costly duplication (people and resources), but as complimentary services (O’Malley, 2010) and thus support their overall business and collaborative strategy. All the private hospitals were certificated to appropriate standards in Thailand and some (4) were even certificated to external overseas organisations such as JCI. Consequently, this illustrated the overarching managerial/marketing approach to connect with patients at home and abroad and to provide medical services that were appealing to doctors, nurses and patients (Lake *et al*. 2003). For all private hospitals, it would appear that upto date technology in many areas of expertise were utilised, providing opportunities for staff development (Lake, ibid) marketing communications, more differentiated patient medical needs and greater supplementary facilities utilisation (Lesser and Brewster, 2001). However, the impacts of this hospital strategy may result in lower patient volumes (Chassin and Galvin, 1998) and does little to help doctors and nursing training requirements.

Q2. In what ways do hospital services provide for international patient health requirements both in Thailand and overseas?

Most of the Thai private hospitals sampled in this research have a wide and what appears to be an appropriate range of medical service provision. However, arrangements appear to be in place to meet the needs of most, but not all, of those potentially using the service. Nevertheless, this provision also varies between the hospitals - but hospital management appear to maintain competitive levels of provision and also management targets specific medical and clinical centres/units such as Children’s centres or Teenage Psychology units (over 30 such specialised centres/units available at most private hospitals in the sample) - e.g. www.bumrungrad.com. Hospital processes appeared to be developed very quickly and routinised to ensure appropriate linkages to new medical service provision that included the management of facilities, medication, staff and ancillary personnel; as well as ensuring patient accessibility and availability at the first point of contact. This may be better attenuated with the use of BPR (Gabel *et al.*, 1999) and e-health developments (Bliemel and Hassanein, 2004) which is now becoming more common overseas. As hospital technology diversity and scope becomes more technical, hospital management appear to understand that training becomes an issue, not only for hospital technicians, but also for doctors and nurses. However, the future provision of dealing with nuclear or other hazardous medical materials waste may need to be assessed.

It would also appear that patients can expect to receive almost 5* hotel-like ancillary services which includes food prepared by leading Chef’s and shopping services. Thus illustrating changes to managed care provision (Lesser and Brewster, 2000). Management further appear to understand the implications and issues involved in communicating more effectively with patients (Hargraves and Trude, 2002) and utilise all available media channels, using appropriate language and personal support mechanisms.

Q3. What are the reasons for the choices that private hospital management make to provide hospital services to medical tourists?

One of the main outcomes of the discussions with hospital management was that initially the private hospital targeted home-based patients and the development of marketing approaches overseas sought more involved assets management because private health expenditure had increased (Chee, 2007). This raised the issues of providing for a range of financial offerings - related to home credit-cards and banks was perceived as needing to be developed - especially for overseas patients as most payments were expected to be in cash. The capability, training and availability of experienced staff has made management focus attention on helping patients through an enhanced service-ethic encapsulating appropriate medical treatments where individuals become certificated following training and hospital protocols. This was especially for protocols requiring meticulous medical attention such as viral/bacterial infections, older long-term patients and children.

Investments in more enhanced personnel training and developments and the integration of technology and hospital processes appears to impact on hospital management as a pragmatic solution to patient requirements. This follows on from Hart, Shleifer and Vishny (1997), who indicated that since private health service providers have well-defined control rights, they have [a] strong incentive to invest in innovations. Such technical innovations and their positive impact on clinical outcomes have been reported elsewhere (Sox *et al.*, 1989). Technology is therefore a way for hospital management to drive such innovativeness and together with a enhanced service-ethic and quality to lure new patients (Businessweek.com, 2005) it appears to be a successful and actively encouraging health business model for Thailand.

Finally, the major outcomes of this research that inform hospital policy can be seen in Table 2, below as characterised through the research questions and major themes.

## 5. Conclusions

Medical tourism has been identified by some researchers as potentially beneficial to the Thai economy and in line with this rhetoric has been an increase in private hospitals whose main focus is to accommodate middle-upper class Thai’s and foreigners. However, medical tourism has not been clinically defined in terms of the economic impact as there is little data that directly links patients with medical tourist notions. Nevertheless, it would appear that a number of private hospitals have a management structure, facilities, personnel and the marketing acumen to adapt to changing medical demands located overseas.
